# Supplementary material for: Metabolomic Predictors of Dysglycemia in Two U.S. Youth Cohorts
Source: Metabolites. 2022 Apr 29;12(5):404. doi: 10.3390/metabo12050404 (PMC9147862; doi:10.3390/metabo12050404)
Supplement: Supplementary file 1 [file metabolites-12-00404-s001.zip › metabolites-1700435-supplementary.pdf]

Supplemental Table S1. Background characteristics of 265 Project Viva youth.

	Mean ± SD or % (N)
**Sociodemographic characteristics**	
Female sex, % (N)	47.6% (126)
Hispanic ethnicity, % (N)	5.3% (14)
Family history of type 2 diabetes, % (N)	0.8% (2)
In utero exposure to gestational diabetes	3.4% (9)
**Characteristics at baseline (age ~10 y)**	
Age (years)	12.9 ± 0.6
Body mass index (BMI) z-score [a]	0.67 ± 1.26
Waist circumference (cm)	73.7 ± 12.0
Fasting glucose (mmol/L)	5.0 ± 0.5
Fasting insulin (uU/mL)	14.2 ± 9.2
Total cholesterol (mg/dL)	155.9 ± 26.9
Triglycerides (mg/dL)	67.1 ± 29.9
Low density lipoprotein (LDL; mg/dL)	86.1 ± 23.5
High density lipoprotein (HDL; mg/dL)	56.4 ± 13.4
Tanner stage for pubic hair development >2	84.2% (223)

a According to the World Health Organization (WHO) growth reference for children 5-19 years of age.

Supplemental Table S2. Metabolites with factor loadings within the top 10% of the first factor of reduced rank regression (RRR).

Girls		Boys	
**Identity**	**#Times in Top 10%**	**Identity**	**#Times in Top 10%**
glutamine	4	quinolinate	5
citrate	4	2'-deoxyuridine	5
N-acetylvaline	4	malate	5
myristate (14:0)	4	glutamate	4
margarate (17:0)	4	sarcosine	4
phenylalanine	4	serine	4
kynurenate	4	lactate	4
chenodeoxycholate	4	leucine	4
ornithine	4	N-acetylvaline	4
cystine	4	margarate (17:0)	4
serine	4	caprate (10:0)	4
adenine	4	N-formylmethionine	4
orotate	4	orotate	4
Succinate	4	beta-alanine	4
aspartate	3	tryptophan	4
asparagine	3	tyrosine	4
cortisol	3	thyroxine	4
cortisone	3	arginine	4
creatinine	3	urea	4
glycine	3	aspartate	3
glycerate	3	asparagine	3
trans-urocanate	3	cortisol	3
isoleucine	3	creatinine	3
3-hydroxyisobutyrate	3	salicylate	3
arachidate (20:0)	3	glutamine	3
stearate (18:0)	3	glycine	3
erucate (22:1n9)	3	biliverdin	3
lysine	3	histidine	3
N6,N6,N6-trimethyllysine	3	trans-urocanate	3
heptanoate (7:0)	3	3-hydroxybutyrate (BHBA)	3
hypotaurine	3	3-hydroxyisobutyrate	3
methionine	3	palmitate (16:0)	3
3-hydroxy-3-methylglutarate	3	pentadecanoate (15:0)	3
nicotinamide	3	myristate (14:0)	3
pantothenate	3	erucate (22:1n9)	3
5-methylthioadenosine (MTA)	3	cystine	3
arachidonate (20:4n6)	3	hypotaurine	3
inosine	3	N-acetylmethionine	3
5,6-dihydrothymine	3	taurine	3
3-aminoisobutyrate	3	pantothenate	3
beta-alanine	3	phenylpyruvate	3
uridine	3	arachidonate (20:4n6)	3
ursodeoxycholate	3	inosine	3
cholesterol	3	urate	3
arginine	3	dihydroorotate	3
urea	3	3-aminoisobutyrate	3
proline	3	deoxycholate	3
retinol (Vitamin A)	3	ursodeoxycholate	3
alanine	2	cholesterol	3

N-acetylalanine	2	citrate	3
salicylate	2	kynurenate	3
glutarate (C5-DC)	2	ornithine	3
gamma-glutamylglutamine	2	proline	3
gamma-glutamyltyrosine	2	retinol (Vitamin A)	3
glutamate	2	alanine	2
5-oxoproline	2	glutarate (C5-DC)	2
threonine	2	gamma-glutamyltyrosine	2
sarcosine	2	5-oxoproline	2
lactate	2	threonine	2
biliverdin	2	myo-inositol	2
histidine	2	N-acetylleucine	2
N-acetylleucine	2	stearate (18:0)	2
valine	2	pipecolate	2
palmitate (16:0)	2	methionine	2
nonadecanoate (19:0)	2	4-acetamidobutanoate	2
pentadecanoate (15:0)	2	linoleate (18:2n6)	2
pipecolate	2	chenodeoxycholate	2
caprate (10:0)	2	adenine	2
laurate (12:0)	2	guanosine	2
N-acetylmethionine	2	5,6-dihydrothymine	2
cysteine	2	uridine	2
taurine	2	alpha-ketoglutarate	2
quinolinate	2	succinate	2
phenylpyruvate	2	fumarate	2
4-acetamidobutanoate	2	N-acetylalanine	1
linoleate (18:2n6)	2	cortisone	1
allantoin	2	gluconate	1
urate	2	gamma-glutamylglutamine	1
guanosine	2	glycerate	1
uracil	2	valine	1
alpha-ketoglutarate	2	arachidate (20:0)	1
malate	2	heptanoate (7:0)	1
fumarate	2	laurate (12:0)	1
alpha-tocopherol	2	cysteine	1
serotonin	2	nicotinamide	1
tyrosine	2	phenylalanine	1
vanillylmandelate (VMA)	2	5-methylthioadenosine (MTA)	1
gluconate	1	allantoin	1
myo-inositol	1	uracil	1
3-hydroxybutyrate (BHBA)	1	alpha-tocopherol	1
leucine	1	vanillylmandelate (VMA)	1
dihydroorotate	1	citrulline	1
2'-deoxyuridine	1		
deoxycholate	1		
tryptophan	1		
citrulline	1		

Supplemental Table S3. Associations of metabolites measured at baseline (age ~10 y) with natural-log transformed baseline fasting glucose at follow-up (age ~16 y) among 197 boys in the EPOCH Study.

	**Associations of metabolites at baseline (age 10 y) with ln-fasting glucose at follow-up (age 16 y)**			
	**Unadjusted (*n* = 197)**		**Model 1 (*n* = 197)**	
	**β (95% CI)**	***P***	**β (95% CI)**	***P***
**Boys (*n* = 197)**				
Leucine	0.13 (-0.02, 0.28)	0.10	0.17 (0.01, 0.33)	0.04
Glutamate	-0.06 (-0.15, 0.03)	0.18	-0.08 (-0.17, 0.02)	0.14
Arginine	0.00 (-0.07, 0.07)	0.94	0.01 (-0.07, 0.08)	0.87
Tryptophan	-0.07 (-0.28, 0.14)	0.52	-0.05 (-0.26, 0.16)	0.64
Margarate (17:0)	0.08 (-0.03, 0.18)	0.12	0.10 (-0.01, 0.20)	0.07
Lactate	0.00 (-0.09, 0.08)	0.95	-0.01 (-0.10, 0.08)	0.87
N-Acetylvaline	0.04 (-0.16, 0.24)	0.68	0.05 (-0.15, 0.25)	0.61
Malate	0.02 (-0.08, 0.11)	0.69	0.01 (-0.09, 0.11)	0.84
Caprate (10:0)	-0.05 (-0.16, 0.05)	0.30	-0.05 (-0.15, 0.06)	0.36
Urea	-0.02 (-0.16, 0.13)	0.80	-0.01 (-0.16, 0.14)	0.92
Orotate	0.01 (-0.08, 0.10)	0.83	0.01 (-0.09, 0.10)	0.89
Thyroxine	0.00 (-0.16, 0.17)	0.98	0.01 (-0.16, 0.18)	0.94
N-Formylmethionine	0.05 (-0.08, 0.19)	0.46	0.05 (-0.09, 0.19)	0.47
Sarcosine	0.00 (-0.10, 0.10)	0.96	-0.01 (-0.11, 0.09)	0.84
Quinolinate	-0.04 (-0.12, 0.04)	0.29	-0.05 (-0.13, 0.02)	0.18
Tyrosine	0.05 (-0.15, 0.26)	0.61	0.08 (-0.13, 0.28)	0.47
2'-Deoxyuridine	0.04 (-0.04, 0.12)	0.35	0.05 (-0.03, 0.15)	0.24
Beta-alanine	0.00 (-0.12, 0.13)	0.95	0.01 (-0.12, 0.13)	0.94
Serine	0.00 (-0.16, 0.16)	0.97	0.02 (-0.15, 0.18)	0.83

Model 1: Adjusted for age in quartiles at baseline, difference in age between baseline and follow-up, and Hispanic ethnicity.

Supplemental Table S4. Associations of metabolites measured at baseline (age ~10 y) with natural-log transformed baseline fasting glucose at follow-up (age ~16 y) among 194 girls in the EPOCH Study.

	**Associations of metabolites at baseline (age ~10 y) with ln-fasting glucose at follow-up (age ~16 y)**			
	**Unadjusted (*n* = 194)**		**Model 1 (*n* = 194)**	
	**β (95% CI)**	***P***	**β (95% CI)**	***P***
**Girls (*n* = 194)**				
Glutamine	0.02 (-0.05, 0.09)	0.53	0.03 (-0.04, 0.10)	0.40
Citrate	-0.09 (-0.24, 0.05)	0.21	-0.10 (-0.25, 0.05)	0.21
N-acetylvaline	0.15 (-0.17, 0.47)	0.36	0.17 (-0.16, 0.49)	0.32
Myristate (14:0)	0.09 (-0.05, 0.22)	0.20	0.11 (-0.03, 0.25)	0.13
Margarate (17:0)	0.13 (-0.04, 0.30)	0.12	0.14 (-0.04, 0.31)	0.12
Phenylalanine	0.13 (-0.17, 0.42)	0.40	0.06 (-0.23, 0.35)	0.68
Kynurenate	-0.03 (-0.14, 0.10)	0.69	-0.02 (-0.15, 0.11)	0.76
Chenodeoxycholate	0.01 (-0.08, 0.09)	0.86	0.00 (-0.08. 0.08)	0.99
Ornithine	0.12 (-0.08, 0.31)	0.24	0.15 (-0.07, 0.37)	0.17
Cystine	-0.02 (-0.11, 0.06)	0.55	-0.04 (-0.12, 0.04)	0.38
Serine	0.03 (-0.24, 0.30)	0.83	-0.08 (-0.37, 0.21)	0.58
Adenine	0.03 (-0.14, 0.19)	0.76	0.04 (-0.12, 0.20)	0.62
Orotate	-0.16 (-0.29, -0.02)	0.02	-0.14 (-0.28, 0.00)	0.05
Succinate	-0.07 (-0.28, 0.14)	0.49	0.08 (-0.28, 0.13)	0.47

Model 1: Adjusted for age in quartiles at baseline, difference in age between baseline and follow-up, and race/ethnicity.
